# Oncological Safety of Prepectoral Implant-Based Breast Reconstruction After Conservative Mastectomy: Insights from 842 Consecutive Breast Cancer Patients

**DOI:** 10.3390/cancers17060925

**Published:** 2025-03-08

**Authors:** Lorenzo Scardina, Alba Di Leone, Alejandro Martin Sanchez, Cristina Accetta, Liliana Barone Adesi, Ersilia Biondi, Beatrice Carnassale, Sabatino D’Archi, Flavia De Lauretis, Enrico Di Guglielmo, Antonio Franco, Stefano Magno, Francesca Moschella, Maria Natale, Marzia Salgarello, Eleonora Savia, Marta Silenzi, Giuseppe Visconti, Riccardo Masetti, Gianluca Franceschini

**Affiliations:** 1 Multidisciplinary Breast Center, Department of Women, Children and Public Health Sciences, Fondazione Policlinico Universitario Agostino Gemelli IRCCS, 00168 Rome, Italygianluca.franceschini@policlinicogemelli.it (G.F.); 2 Department of Plastic, Reconstructive and Aesthetic Surgery, Department of Science and Health of Women, Children and Public Health, Fondazione Policlinico Universitario Agostino Gemelli IRCCS, 00168 Rome, Italy

**Keywords:** implant-based breast reconstruction, prepectoral reconstruction, nipple-sparing mastectomy, skin-sparing mastectomy, skin-reducing mastectomy, oncological outcomes, local recurrence

## Abstract

Background: Implant-based breast reconstruction (IBBR) following conservative mastectomy is the most common approach for women undergoing breast cancer surgery. The aim of this study was to compare the oncological outcomes of conservative mastectomy combined with prepectoral IBBR to the subpectoral technique. Methods: The clinical and demographic data of consecutive breast cancer patients who underwent conservative mastectomy with either prepectoral or subpectoral IBBR between January 2018 and December 2023 were retrospectively analyzed. The primary outcome was the impact of conservative mastectomy with prepectoral IBBR on local recurrence-free survival (LRFS). Secondary outcomes included distant disease-free survival (DDFS) and overall survival (OS). Results: A total of 842 women (with a median age of 46 years and a range of 20–79 years) were included in the study. Of these, 648 patients (77.0%) underwent prepectoral IBBR, while 194 (23.0%) received subpectoral IBBR. The median follow-up was 32 months (3–74). Locoregional relapse occurred in 19 patients (2.9%) in the prepectoral group and 14 (7.2%) in the subpectoral group. Distant metastases were observed in 21 (3.2%) patients in the prepectoral group and 11 (5.7%) in the subpectoral group. Deaths were reported in eight patients (1.2%) in the prepectoral group and five (2.6%) in the subpectoral group. There were no statistically significant differences between the two groups in terms of the LRFS, DDFS, and OS (*p* = 0.676; *p* = 0.994; *p* = 0.940, respectively). Conclusions: Our study indicates that conservative mastectomy combined with prepectoral IBBR produces similar results to those of the subpectoral approach, with no significant differences in LRFS, DDFS, and OS.

## Simple Summary

In this study, we retrospectively analyzed clinical and demographic data from 842 consecutive breast cancer patients who underwent conservative mastectomy with prepectoral implant-based breast reconstruction (IBBR) from January 2018 to December 2023 at Fondazione Policlinico Universitario Agostino Gemelli IRCCS in Rome. Several retrospective studies and meta-analyses have demonstrated that prepectoral IBBR is a safe surgical option, yielding clinical outcomes comparable to the subpectoral approach. However, there is limited evidence regarding residual breast tissue after conservative mastectomy, which remains a potential risk for local recurrence. The oncological safety of conservative mastectomy, when combined with prepectoral IBBR, remains a topic of ongoing discussion. This is the first study in the literature with such a large sample size comparing the oncological outcomes of conservative mastectomy combined with prepectoral IBBR to the subpectoral technique.

## 1. Introduction

Over the past two decades, the surgical treatment of breast cancer has undergone rapid evolution aiming to provide effective oncological control while improving cosmetic outcomes [1]. Conservative mastectomy, including nipple-sparing, skin-sparing, and skin-reducing techniques, has become a standard approach for women who are not candidates for breast-conserving surgery [2].

These procedures aim to remove glandular breast tissue while preserving the healthy skin envelope and offering both oncological safety and esthetic benefits. Implant-based breast reconstruction (IBBR) has further enhanced the quality of life for many patients allowing the restoration of breast shape post mastectomy [3].

Among the IBBR techniques, subpectoral placement, where the implant is placed beneath the pectoralis major muscle, has traditionally been the most common [4].

However, the prepectoral approach, in which the implant is positioned above the muscle, has gained significant attention in recent years. This technique offers several advantages, including the avoidance of muscle dissection, reduced postoperative pain, and faster recovery [5,6]. It also leads to fewer animation deformities compared to subpectoral reconstruction, where the muscle’s movement during arm motion can distort the implant. These factors, combined with improved cosmetic results and patient satisfaction, have contributed to the rising popularity of prepectoral IBBR [7].

Despite these benefits, concerns about the oncological safety of prepectoral reconstruction remain a topic of ongoing debate. One key issue is the potential risk of local recurrence due to residual breast tissue that may remain after conservative mastectomy. Although the procedure aims to preserve well-vascularized and appropriately sized skin flaps to reduce surgical complications, even small amounts of residual tissue could contribute to an increased risk of recurrence. This is a critical issue that has yet to be fully addressed in the context of prepectoral reconstruction [8,9].

Several algorithms and selection criteria have been proposed to identify the candidates for this technique [10,11].

Therefore, we assume that prepectoral IBBR following conservative mastectomy offers benefits in terms of quality of life and patient satisfaction, as previously reported by some authors [12,13,14].

Although preliminary studies have suggested that prepectoral IBBR is a safe and effective option, comprehensive data comparing the oncological outcomes to the traditional subpectoral approach remain limited. The primary unresolved question is whether the prepectoral technique can achieve comparable oncological safety, particularly in terms of locoregional recurrence and distant metastases.

This study seeks to fill this gap by directly comparing the oncological safety of prepectoral IBBR with the subpectoral approach in a cohort of 842 consecutive breast cancer patients. By analyzing key oncological endpoints, we aim to provide definitive evidence on the safety of prepectoral IBBR following conservative mastectomy.

With a large sample size and an adequate follow-up period, the study offers the opportunity to evaluate the long-term oncological outcomes of these two reconstruction techniques. By addressing the oncological safety of prepectoral IBBR in comparison to the subpectoral technique, our findings provide valuable insights into whether prepectoral IBBR can be considered a viable and safe alternative for patients undergoing conservative mastectomy.

Despite a significant body of literature supporting the oncological safety of conservative mastectomy with subpectoral IBBR [15,16,17], there is still a lack of high-level evidence regarding the oncological outcomes of prepectoral IBBR, making this a controversial topic [18].

The aim of this study is to demonstrate that prepectoral IBBR following conservative mastectomy is oncologically safe and provides adequate surgical margins while reducing complications.

## 2. Materials and Methods

This study was a retrospective review of 842 consecutive breast cancer patients who underwent conservative mastectomy with either prepectoral or subpectoral IBBR at the Fondazione Policlinico Universitario Agostino Gemelli IRCCS in Rome, Italy, between January 2018 and December 2023. The study was approved by the Institutional Review Board (Lazio-3 Ethics Committee, 14 December 2023, ID number: 6308), and all patients provided informed consent for their participation.

### 2.1. Study Population

The study cohort consisted of adult women diagnosed with breast cancer who underwent conservative mastectomy with immediate IBBR. Conservative mastectomy was defined as nipple-sparing, skin-sparing, or skin-reducing mastectomy. The inclusion criteria were as follows: patients who underwent oncologic conservative mastectomy and immediate IBBR and with a TNM stage of I–III. The exclusion criteria were the following: previous radiotherapy, two-stage IBBR or other type of reconstruction planned, active smokers, and significant comorbidity (diabetes, obesity, connective tissue disease).

Clinical data, including age, tumor characteristics (histological type and disease stage), reconstruction type, adjuvant therapies, disease status at the last follow-up, and postoperative surgical complications, were recorded. Both groups were compared for demographic and clinical characteristics, including tumor size and lymph node involvement.

### 2.2. Surgical Procedures

All surgeries were performed by experienced breast and plastic surgeons following standardized protocols. In the prepectoral group, the implant was placed above the pectoralis major muscle, directly beneath the skin and tissue flaps, with no muscle dissection. In the subpectoral group, the implant was placed beneath the pectoralis major muscle. Conservative mastectomy was routinely offered to patients who were unable to achieve clear margins with breast-conserving surgery.

An innovative multidisciplinary approach, known as the ROME (radiological and oncoplastic multidisciplinary evaluation) model, was used at our institution. The ROME framework integrates radiologists, breast surgeons, oncoplastic specialists, oncologists, radiation oncologists, and geneticists to assess each patient’s anamnestic, morphological, functional, and oncological criteria. This collaborative evaluation was used to determine the most appropriate mastectomy and reconstruction technique for each patient [19].

### 2.3. Follow-Up and Outcomes

The primary endpoint of this study was locoregional recurrence-free survival (LRFS), defined as the time from surgery to the first locoregional recurrence. Secondary endpoints included distant disease-free survival (DDFS) and overall survival (OS). DDFS was defined as the time from surgery to the first distant metastasis, while OS was the time from surgery to death from any cause.

Patients were followed up regularly with clinical examinations and imaging (including mammograms, ultrasound, or MRI) at 6-month intervals, with additional systemic evaluation based on disease stage.

### 2.4. Statistical Analysis

Descriptive statistics for the variables of interest in relation to the two groups were initially reported. Since all variables were categorical, absolute frequencies and column percentages were used. A univariate analysis was then performed using chi-square tests to assess potential associations between these variables and the two groups. Logistic regression was employed to identify which variables significantly influenced the likelihood of belonging to a particular group. Additionally, separate Kaplan–Meier curves were generated for the three events (local recurrence, distant disease recurrence, and death) for each group, and the corresponding hazard ratios were calculated. All statistical analyses were conducted using IBM SPSS v.28 software, with a significance threshold set at *p* < 0.05.

## 3. Results

This study included 842 women, with a median age of 46 years (range: 20–79). In total, 1101 mastectomies were performed, with 648 patients (77.0%) undergoing conservative mastectomy followed by prepectoral IBBR and 194 patients (23.0%) undergoing subpectoral IBBR. The median follow-up period was 32 months (range: 3–74), with a median of 27 months for the prepectoral group and 50 months for the subpectoral group.

### 3.1. Oncological Staging

The majority of patients (483, 57.4%) were diagnosed with stage I disease, with 371 (57.3%) in the prepectoral group and 112 (57.7%) in the subpectoral group. Stage II disease was diagnosed in 290 patients (34.4%), with 224 (34.6%) in the prepectoral group and 66 (34.0%) in the subpectoral group. Stage III disease was observed in 69 patients (8.2%), with 53 (8.1%) in the prepectoral group and 16 (8.3%) in the subpectoral group. The clinicopathological characteristics for each surgical group are provided in Table 1.

### 3.2. Surgical Technique

Of the total cohort, 761 patients (90.4%) underwent nipple-sparing mastectomy, with 583 in the prepectoral group and 178 in the subpectoral group. Skin-sparing mastectomy was performed in 73 patients (8.7%) including 58 in the prepectoral group and 15 in the subpectoral group. Additionally, eight patients (0.9%) underwent skin-reducing mastectomy, all in the prepectoral group.

Regarding the mastectomy type, 583 patients (69.3%) had a unilateral mastectomy, and 259 (30.7%) underwent bilateral mastectomy. Contralateral symmetrization was required for 105 patients (22.3%) in the prepectoral group and 75 patients (66.9%) in the subpectoral group. In terms of surgical incision, 716 patients (85.1%) had a radial incision in the outer quadrants, 83 (9.8%) received a complete periareolar incision, 19 (2.3%) had an inframammary fold incision, 15 (1.8%) underwent a transaxillary incision, 8 (0.9%) had an incision following the skin-reducing mastectomy pattern, and 1 (0.1%) had an incision from the upper quadrants.

### 3.3. Neoadjuvant and Adjuvant Treatments

Neoadjuvant chemotherapy was given to 293 patients (34.8%), with 223 (34.4%) in the prepectoral group and 70 (36.1%) in the subpectoral group. Postoperative radiotherapy was administered to 226 patients (26.8%), including 181 (27.9%) in the prepectoral group and 45 (23.2%) in the subpectoral group. These treatments were part of the personalized multidisciplinary care plan for each patient.

### 3.4. Surgical Complications

Surgical complications requiring intervention were observed in 30 patients (3.6%), including wound dehiscence, flap necrosis, NAC necrosis, hematoma, and infections. Of these, 24 patients (3.7%) were in the prepectoral group and 6 patients (3.1%) were in the subpectoral group. These complications led to device replacement in seven cases (1.1%) in the prepectoral group and eleven cases (5.7%) in the subpectoral group. The overall complication rates were not significantly different between the groups, as detailed in Table 2.

### 3.5. Oncological Outcomes

**Figure 1:**
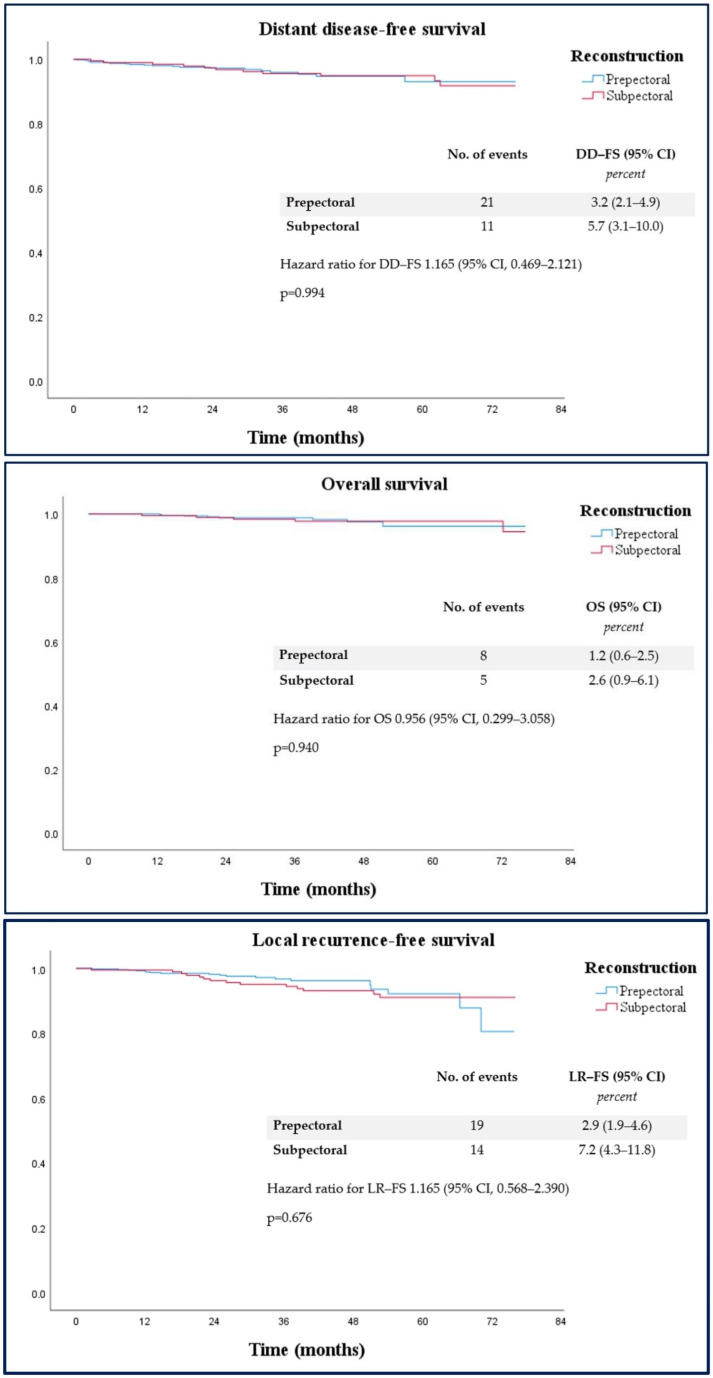
Kaplan–Meier curves showing local recurrence-free survival, distant disease-free survival, and overall survival of breast cancer patients treated with conservative mastectomy and prepectoral vs. subpectoral implant-based breast reconstruction.

In terms of oncological outcomes, 19 patients (2.9%) in the prepectoral group experienced locoregional recurrences, compared to 14 (7.2%) in the subpectoral group. Distant metastases occurred in 21 patients (3.2%) in the prepectoral group and 11 (5.7%) in the subpectoral group. Additionally, eight deaths (1.2%) were recorded in the prepectoral group, while five deaths (2.6%) occurred in the subpectoral group (Table 3). These findings suggest a significantly lower rate of locoregional recurrence in the prepectoral group. Kaplan–Meier survival curves and the corresponding hazard ratios for LRFS, DDFS, and OS showed no statistically significant differences between the two groups (*p* = 0.676 for LRFS, *p* = 0.994 for DDFS, *p* = 0.940 for OS) (Figure 1).

## 4. Discussion

This study offers significant insights into the oncological safety of prepectoral IBBR compared to the traditional subpectoral technique following conservative mastectomy in breast cancer patients. The findings not only emphasize the oncological equivalence of the two techniques but also highlight the critical role of surgical technique and careful preoperative patient selection in achieving optimal outcomes. With a large cohort of 842 women and a median follow-up of 32 months, our results contribute to the growing body of evidence supporting prepectoral IBBR as a safe and effective option for breast cancer patients.

Conservative mastectomy with prepectoral IBBR has dramatically increased over the past five years and has achieved success in the breast surgery scenario, even in the absence of any high level of evidence [20,21,22].

**Figure 2:**
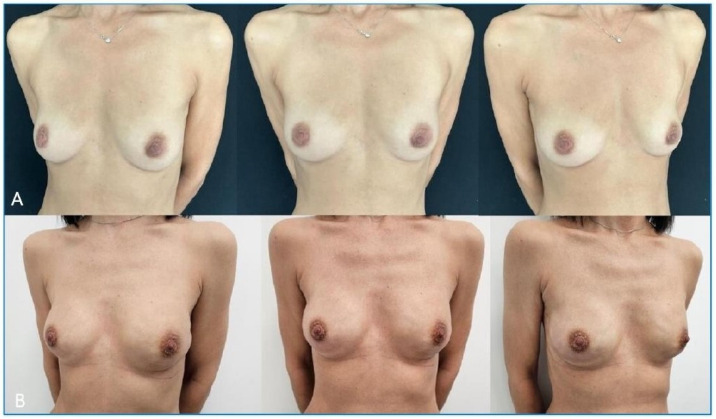
(**A**) 45-year-old preoperative bilateral breast cancer patient. (**B**) Six-month postoperative pictures after bilateral nipple-sparing mastectomy and prepectoral IBBR at Fondazione Policlinico Universitario Agostino Gemelli IRCCS in Rome.

According to a literature review on new insights on IBBR after conservative mastectomy, many authors have demonstrated that the prepectoral approach is safe and feasible, with excellent short-term esthetic results and patient satisfaction [23,24] (Figure 2).

In a large meta-analysis, Nolan et al. demonstrated the safety of immediate prepectoral breast reconstruction after nipple-sparing mastectomy, compared with submuscular techniques [25].

Thus, like many authors, we consider the optimal surgical outcomes of prepectoral reconstruction after conservative mastectomy to be well-established [26,27]. Our analysis also confirmed these findings. Specifically, with respect to NAC necrosis, wound dehiscence, skin flap necrosis, hematoma, and infection, we not only observed complication rates significantly lower than those reported in other studies [28] but also found no statistically significant differences between the prepectoral and submuscular groups.

Notably, the need for implant replacement was higher in the subpectoral group (5.7%) compared to the prepectoral group (1.1%), which may be linked to the muscle dissection involved in the subpectoral technique and its potential to cause more postoperative complications.

The primary goal of this study was to compare the oncological outcomes of prepectoral versus subpectoral IBBR, focusing on LRFS, DDFS, and OS. The results demonstrate that prepectoral IBBR yields oncological outcomes comparable to the subpectoral approach, with no significant differences in LRFS, DDFS, and OS. These findings are particularly reassuring, given the ongoing concerns in the literature about the potential for local recurrence after conservative mastectomy, particularly related to residual breast tissue.

A key factor in ensuring these favorable oncological outcomes is the precision of the surgical technique. To eliminate the risk of residual glandular tissue, a conservative mastectomy must be performed with accuracy, respecting anatomical planes. The technique involves carefully separating skin flaps from glandular tissue along Camper’s fascia and ensuring complete tissue removal. This meticulous dissection, alongside accurate palpation of the skin flaps and preoperative digital mammography to assess the flap quality, is central to maintaining oncological safety [29,30].

The difference between conservative mastectomy and traditional mastectomy lies primarily in the preservation of tissue and the surgical approach. A conservative mastectomy aims to preserve more of the skin of the breast, leaving the skin flaps intact or minimally altered to allow for a better cosmetic result, particularly for prepectoral implant placement. The underlying breast tissue is removed, but it allows for the possibility of preserving a portion of the breast skin, which is important for future reconstruction. In the traditional mastectomy, the goal is to remove as much tissue as possible to ensure complete oncological safety and it does not necessarily require preserving anatomy by maintaining the subcutaneous tissue. The main goal of this technique is to preserve skin integrity and ensure an optimal esthetic outcome for reconstruction, but this does not mean that the subcutaneous tissue must always be retained. Therefore, in most cases, this approach allows for a submuscular prosthetic reconstruction.

Interestingly, while the locoregional recurrence rate was lower in the prepectoral group (2.9%) compared to the subpectoral group (7.2%), the difference was not statistically significant. This may be due to the study’s median follow-up period of 32 months, which may not have been sufficient to detect significant long-term differences in recurrence rates. Similarly, the distant metastasis rates were lower in the prepectoral group (3.2%) compared to the subpectoral group (5.7%), but the differences did not reach statistical significance. Nonetheless, these results suggest that when performed correctly, prepectoral IBBR does not compromise oncological safety.

Additionally, on patients with loco-regional recurrence who underwent surgical treatment, a local wide excision or radical mastectomy with the removal of the implant were performed [31]. In each case, every single patient underwent systemic staging with a CT scan and bone scintigraphy or PET-CT, which excluded the presence of distant metastases. All cases were discussed at the multidisciplinary meeting. The surgical technique used in the case of local recurrence was as simple as possible, the skin incision above the lesion was primarily chosen, and the periprosthetic capsule was not incised, when possible.

An important factor contributing to the successful outcomes observed in our study is the preoperative patient evaluation using the ROME model. This comprehensive multidisciplinary approach ensures that only patients most likely to benefit from prepectoral IBBR are selected. By evaluating the patients’ anatomy, clinical history, and other relevant factors, the ROME model optimizes both oncological and esthetic outcomes, minimizes complications, and prevents incomplete tissue removal, which are critical for ensuring oncological safety. Our data further support that when preoperative planning and surgical techniques are followed appropriately, prepectoral reconstruction does not increase the risk of local recurrence.

In addition to oncological outcomes, patient satisfaction, and esthetic results are crucial factors in evaluating the success of breast reconstruction. The prepectoral technique is associated with reduced postoperative pain, faster recovery, and fewer animation deformities, making it an increasingly preferred choice for patients. These factors not only improve patient comfort but also contribute to better functional and esthetic outcomes. Moreover, the lower rates of implant-related complications in the prepectoral group further reinforce its advantages.

In patients who require adjuvant radiotherapy, fat grafting can be a valuable adjunct, offering improved esthetics, soft tissue support, and increased patient satisfaction. However, careful patient selection and optimization of the technique are essential to achieve the best outcomes, especially considering the potential impact of radiation on tissue quality and fat graft survival [32,33].

However, there are limitations to consider. The retrospective design of the study introduces potential biases, such as selection bias, which may affect the comparison between the two groups. Since the study was not randomized, patients who underwent one type of reconstruction may have had different baseline characteristics (e.g., tumor size, stage, or comorbidities) that could influence the outcomes.

## 5. Conclusions

In conclusion, prepectoral IBBR following conservative mastectomy demonstrates oncological outcomes comparable to the subpectoral approach, with no significant differences in LRFS, DDFS, and OS. Although the findings are encouraging, longer follow-ups and continued research are necessary to comprehensively assess the long-term safety and efficacy of prepectoral reconstruction. These results support the ongoing exploration of prepectoral IBBR as a safe and effective option for patients undergoing conservative mastectomy.

## Figures and Tables

**Table 1:** Comparison between the patients’ clinical characteristics. NSM: Nipple-sparing mastectomy; SSM: skin-sparing mastectomy; SRM: skin-reducing mastectomy; CDI: invasive ductal carcinoma; CLI: invasive lobular carcinoma; CDIS: ductal carcinoma in situ; NAD: neoadjuvant chemotherapy; RT: radiation therapy; VP: pathogenic variant.

Characteristics of Overall Sample (N = 842)	Prepectoral (N = 648; 77.0%)	Subpectoral (N = 194; 23.0%)	Univariate Analysis	Multivariable Analysis
			*p*-Value	*p*-Value; OR (95% CI)
**Demographics**				
Age (years)			**<0.001**	
- ≤50	414 (63.9%)	161 (83.0%)		
- >50	234 (33.1%)	33 (17.0%)		**<0.001; 0.418 (0.265–0.660)**
**Surgery**			0.287	
- NSM	583 (90.0%)	178 (91.8%)		
- SSM	57 (8.8%)	16 (8.2%)		0.226; 0.347 (0.062–1.927)
- SRM	8 (1.2%)	0 (0.0%)		>0.999; 0.00 (0.00-)
**Surgery**			**<0.001**	
- Monolateral	470 (72.5%)	113 (58.3%)		
- Bilateral	178 (27.5%)	81 (41.7%)		**<0.001; 4.479 (2.746–7.306)**
**Incision**			**<0.001**	
- External quadrants	555 (85.7%)	161 (83.0%)		
- Superior quadrants	1 (0.2%)	0 (0.0%)		>0.999; 0.00 (0.00-)
- Inframammary fold	17 (2.6%)	2 (1.0%)		0.187; 0.359 (0.078–1.642)
- Periareolar	65 (10.0%)	18 (9.3%)		0.134; 3.485 (0.681–17.834)
- SRM	8 (1.2%)	0 (0.0%)		>0.999; 0.00 (0.00-)
- Transaxillary	2 (0.3%)	13 (6.7%)		**<0.001; 15.536 (3.310–72.915)**
**Contralateral surgery**			**<0.001**	
- None	365 (56.3%)	38 (19.6%)		
- Additive implant	9 (1.4%)	19 (9.7%)		**<0.001; 4.306 (2.784–6.660)**
- Reductive mastoplasty	96 (14.8%)	56 (28.9%)		**<0.001; 21.969 (8.918–54.118)**
- Bilateral mastectomy	178 (27.5%)	81 (41.8%)		**<0.001; 5.206 (3.159–8.580)**
**Tumor sub-type**			0.747	
- CDI	475 (73.3%)	143 (73.7%)		
- CLI	76 (11.7%)	26 (13.4%)		0.158; 1.496 (0.855–2.617)
- CDIS	83 (12.8%)	20 (10.3%)		**0.041; 0.493 (0.250–0.972)**
- Others	14 (2.2%)	5 (2.6%)		0.827; 0.879 (0.275–2.803)
**T**			0.495	
- 0	79 (12.2%)	29 (14.9%)		
- is	77 (11.9%)	19 (9.8%)		
- 1mi	32 (4.9%)	8 (4.1%)		
- 1a	51 (7.8%)	13 (6.7%)		
- 1b	69 (10.7%)	25 (12.4%)		
- 1c	153 (23.6%)	57 (29.4%)		
- 2	165 (25.5%)	40 (20.6%)		
- 3	21 (3.2%)	4 (2.1%)		
- 4	1 (0.2%)	0 (0.0%)		
**N**			0.433	
- 0	410 (63.2%)	114 (58.8%)		
- ITC	13 (2.0%)	4 (2.0%)		
- 1mi	34 (5.3%)	8 (4.2%)		
- 1	139 (21.5%)	52 (26.8%)		
- 2	39 (6.0%)	9 (4.6%)		
- 3	13 (2.0%)	7 (3.6%)		
**STAGE**			0.990	
- I	371 (57.3%)	112 (57.7%)		
- II	224 (34.6%)	66 (34.0%)		0.774; 1.067 (0.687–1.656)
- III	53 (8.1%)	16 (8.3%)		0.317; 1.464 (0.694–3.091)
**NAD**			0.669	
- Yes	223 (34.4%)	70 (36.1%)		0.302; 0.793 (0.511–1.231)
- No	425 (65–6%)	124 (63.9%)		
**RT**			0.192	
- Yes	181 (27.9%)	45 (23.2%)		0.481; 0.836 (0.508–1.377)
- No	467 (72.1%)	149 (76.8%)		
**Multifocality**			**0.011**	
- Yes	216 (33.3%)	46 (23.7%)		**0.046; 0.639 (0.411–0.993)**
- No	432 (66.7%)	148 (76.3%)		
**VP**			0.462	
- NO	539 (83.2%)	160 (82.5%)		
- BRCA1	51 (7.9%)	21 (10.8%)		0.431; 0.773 (0.408–1.465)
- BRCA2	32 (4.9%)	8 (4.1%)		0.275; 0.618 (0.260–1.466)
- Others	26 (4.0%)	5 (2.6%)		0.070; 0.372 (0.128–1.082)

**Table 2:** Surgical outcomes.

Any Breast-Related Complication	All pz	Prepec	Subpec	*p*-Value
Minor complications (NAC necrosis, wound dehiscence)	9 (1.1%)	8 (1.2%)	1 (0.5%)	0.393
Major complications (Skin flap necrosis, hematoma, infection)	21 (2.5%)	16 (2.5%)	5 (2.6%)	0.932

**Table 3:** Oncological outcomes.

	Overall	Prepec	Subpec
LRFS rate	33 (3.9%)	19 (2.9%)	14 (7.2%)
DDFS rate	32 (3.8%)	21 (3.2%)	11 (5.7%)
OS rate	13 (1.5%)	8 (1.2%)	5 (2.6%)

## Data Availability

The data presented in this study are available from the corresponding author upon request.

## Abbreviations

The following abbreviations are used in this manuscript:

IBBR	Implant-based breast reconstruction
ROME	Radiological and oncoplastic multidisciplinary evaluation
LRFS	Locoregional recurrence-free survival
DDFS	Distant disease-free survival
OS	Overall survival
